# Deep learning-based automatic segmentation and classification for cervical cancer detection using an improved U-Net and ensemble methods

**DOI:** 10.1038/s41598-026-35299-7

**Published:** 2026-01-13

**Authors:** Betelhem Zewdu Wubineh, Andrzej Rusiecki, Krzysztof Halawa

**Affiliations:** 1 Faculty of Information and Communication Technology, Wroclaw University of Science and Technology, Wroclaw, Poland; 2 Department of Information Technology, College of Engineering and Technology, Wachemo University, Hossana, Ethiopia

**Keywords:** Cervical cancer, Deep learning, Classification, Segmentation, Ensemble learning, RES_DCGAN, Improved U-Net, Cancer, Cell biology, Computational biology and bioinformatics, Medical research, Oncology

## Abstract

Cervical cancer is the fourth leading cause of cancer-related illness and death among women worldwide. The Pap test is a widely used screening technique to detect abnormal cells that can become cancerous. In this research, we proposed a method for automatic segmentation and classification of cervical cancer cell images. The method uses an improved U-Net architecture to segment the image and identify the region of interest (ROI). Following segmentation, we classify the cervical cell type using ResNet50V2 and an ensemble of different pretrained models to enhance performance. We developed several pipelines for cervical cancer detection, including a normal method, with and without RES_DCGAN, before classification and segmentation tasks. The proposed method was evaluated using the Pomeranian, Herlev, and SIPaKMeD datasets. The experimental results showed that whole-cell segmentation achieved 99.53%, 88.95%, and 98.3% accuracy when RES_DCGAN was added before the segmentation. The framework achieved 96% and 95% accuracy for multi-class classification on the Pomeranian and SIPaKMeD datasets, respectively. Additionally, the Herlev dataset scored an accuracy of 91%, while SIPaKMeD achieved 99% for the binary classification of cervical cell types using the ensemble method. In conclusion, the deep learning-based segmentation and classification method demonstrated promising results for cervical cancer detection and can help pathologists diagnose the disease.

## Introduction

Cervical cancer is the fourth most common cause of cancer morbidity and mortality among women worldwide, significantly affecting public health^1^. The prognosis for cervical cancer depends on the stage at which the cancer is detected^2^. However, it is curable at its premalignant stage, making early detection crucial for successful treatment^3^. Early detection of cervical cancer significantly reduces morbidity and mortality, as the disease is slow-growing and progresses through identifiable precancerous lesions^4^. Many deaths related to cervical cancer occur because the disease often goes undetected in its early stages, and patients generally show no symptoms until it has advanced to a more critical terminal phase^5^. The Pap smear, also known as the Papanicolaou test, is a widely used screening technique for preventing cervical cancer by detecting abnormal cells that may become cancerous^2^. This method involves microscopic examination of cervical cells, allowing for timely treatment^1^. However, its accuracy is highly dependent on the clinician’s expertise, making it time-consuming and susceptible to human error, even for experienced doctors^3^. The effectiveness of Pap smear screening is dependent on several factors, including access to healthcare facilities, the quality of screening tests, and the proper diagnosis and treatment of detected lesions. Unfortunately, in many developing countries, Pap test screening services are scarce due to a shortage of trained medical professionals and insufficient resources to support these programs.

Medical image processing and intelligent systems offer a more cost-effective and time-efficient alternative to traditional methods such as Pap smears, colposcopy, and cervicography to analyze malignant cells^6^. Computer-aided diagnosis (CAD) systems are emerging as valuable tools to assist pathologists in detecting and diagnosing cervical cancer^7^. Systematic screening with these systems has the potential to reduce cervical cancer mortality rates by 70% or more^3^. Today, deep learning techniques have become powerful tools for medical image analysis, with numerous methodologies proposed for the segmentation and classification of cervical cancer^8^. From an image processing perspective, accurately identifying and segmenting each cell component: nucleus, cytoplasm, and background, is the first step in extracting relevant information^2,9^. Modern technology further simplifies cervical cancer detection by analyzing changes in the patterns of cervical cells, particularly the color and shape of the nucleus and cytoplasm, as seen in medical images such as Pap smears^10^. These images can provide crucial information about the presence of cervical cancer, helping with early diagnosis and treatment. After the cells are precisely segmented, the next step is the classification phase. The basic characteristics used to classify the stage of cells, such as shape, size, texture, and nucleus-to-cytoplasm ratio, are critical for detecting cervical cancer. Automated classification of these cells can significantly accelerate the diagnosis process and facilitate timely medical intervention^1^.

Several studies have attempted cervical cell segmentation and classification using deep learning, but with notable differences in methodologies, datasets, and scope. Kurnianingsih et al.^11^ applied Mask R-CNN with a VGG-like classifier, focusing on whole-cell segmentation in the Herlev dataset. Sabeena and Gopakumar^12^ used a pyramid scene parsing model to segment images, and employed Naïve Bayes, MLP, random forest, J48, and SVM classifiers to leverage both local and global context features using the Herlev dataset. Battula and Chandana^5^ implemented SegNet with EfficientNet for segmentation and classification, and added handcrafted morphological and texture features for enhanced performance on the Herlev dataset. In contrast, Tripathi et al.^13^ used ResNet152, and Fang et al.^14^ utilized a deep CNN called DeepCELL, focused primarily on classification, without prior segmentation using the SIPaKMeD dataset. More recently, explainable AI (XAI) methods have been explored: one study employed GradCAM, GradCAM++, and Layer-wise Relevance Propagation (LRP) with a VGG16 classifier on the Herlev dataset to improve interpretability^15^, while another introduced EnsembleCAM, combining multiple CAM methods with an XceptionNet classifier to enhance transparency in decision-making^16^. Additionally, study^17^ employed GAN-based augmentation solely for classification, leaving its impact on segmentation unexplored.

While these methods achieved significant results, they often faced limitations, including insufficient multi-scale feature extraction, small-batch training instability, limited generalizability due to conventional augmentation, and a lack of integration between augmentation and segmentation. To address these gaps, our study proposes a novel integrated framework: an improved U-Net architecture with dilated convolutions and group normalization for robust, multi-scale segmentation, combined with RES_DCGAN-based data augmentation applied both before and after segmentation to expand training datasets and enhance model generalizability. Segmented features are classified using ResNet50V2 and ensemble methods across multiple datasets (Pomeranian, Herlev, SIPaKMeD). Unlike prior work, this approach evaluates the impact of RES_DCGAN augmentation on segmentation performance, extending GAN-based augmentation beyond classification. The contributions of this study are as follows.

- Improved U-Net architecture with dilation in the encoder and group normalization in the encoder and decoder to capture multi-scale context and stabilize training.
- Integration of segmentation and classification pipelines to detect cervical cancer.
- Application of RES_DCGAN augmentation before and after segmentation to optimize model performance.
- Evaluation across multiple datasets with different numbers of classes, demonstrating generalizability and robustness.

Accordingly, this study is guided by the following research questions: (1) Can segmentation improve classification performance by isolating meaningful regions of interest in cervical cell images? (2) Does data augmentation using a residual DCGAN (RES_DCGAN) improve the robustness and generalizability of the segmentation model? and (3) Which classification strategies achieve the best performance across different datasets? This study aims to develop a method to segment images into different parts to identify the region of interest and automatically classify cervical cell types. The remainder of this paper is organized as follows. “Results” presents the results, “Discussions” discusses the findings, “Methods” describes the materials and methods used, and “Conclusions” offers concluding remarks.

## Results

This section presents the experimental outcomes of the proposed pipelines for segmenting and classifying cervical cells. The segmentation task aimed to separate the whole cell (cytoplasm and nucleus) from the background, while the classification task categorized cervical cell types using Pomeranian, Herlev, and SIPaKMeD datasets. The first row presents the results for the standard U-Net architecture using the real datasets. The following six rows show the results from the improved U-Net architecture, in which dilation was added, and batch normalization was replaced with group normalization. Because deep learning models involve stochastic processes (e.g., random weight initialization and shuffling of training samples), repeated runs of the same experiment may produce slightly different segmentation scores. However, these variations were minor and did not affect the overall ranking of the methods. For pipelines that share identical segmentation stages, such as Normal, Nor_RES_DCGAN, ROI, and ROI_RES_DCGAN, the segmentation results are expected to be equivalent. Therefore, to avoid redundancy in the tables, only one representative segmentation score is reported for each group of pipelines. This does not alter the interpretation of the results, as the relative performance across methods remains consistent. Similarly, for pipelines using RES_DCGAN before segmentation (RES_DCGAN_Nor and RES_DCGAN_ROI), both pipelines produced nearly identical segmentation metrics. We therefore report one representative segmentation result for clarity. This decision does not affect the comparative conclusions, which focus on differences in the classification stage. However, the classification pipelines differ; so, all six pipeline values are reported in the tables. To evaluate performance, segmentation was assessed using accuracy, precision, recall, Dice coefficient, and intersection over union (IoU). Classification was evaluated using accuracy, precision, recall, and F1-score.

### Pomeranian dataset

The results for all pipelines using the Pomeranian dataset are shown in Table 1, where Acc is accuracy, Pre is precision, Rec is recall, Dice is the dice coefficient, and F1 is the F1-score. All performance metrics are expressed as percentages.

**Table 1:** The result of the study using the Pomeranian dataset.

Methods/pipelines	Segmentation					Classification											
						ResNet50V2				Voting				Ensemble			
	Acc	Pre	Rec	Dice	IoU	Acc	Pre	Rec	F1	Acc	Pre	Rec	F1	Acc	Pre	Rec	F1
Standard U-Net	98.49	94.53	93.09	92.41	98.42	96	97	96	96	89	91	89	88	98	98	98	98
Normal	99.45	97.80	97.71	90.04	99.34	89	89	89	88	92	93	92	91	**96**	**96**	**96**	**96**
Nor_RES_DCGAN						92	93	92	91	86	88	86	82	95	96	95	95
ROI						82	79	82	80	86	89	86	83	85	83	85	83
ROI_RES_DCGAN						82	81	82	81	86	90	86	83	80	77	80	74
RES_DCGAN_Nor	**99.53**	98.09	98.09	90.88	**99.43**	92	92	92	91	87	89	87	84	95	96	95	95
RES_DCGAN_ROI						86	89	86	82	86	88	86	82	86	88	86	82

In Table 1, the results indicate that the standard U-Net achieved 98.49% accuracy, the improved U-Net with real data reached 99.45%, and the model trained on generated data before segmentation achieved the highest accuracy of 99.53%. This demonstrates that the proposed methods yield high accuracy, with slight improvement when RES_DCGAN is applied before segmentation. For classification, the ensemble method consistently outperformed ResNet50V2 and voting, achieving the highest accuracy of 98% in the ensemble method. Segmentation outputs used directly for classification generally performed better than using ROI inputs. For instance, the Normal method achieved an accuracy of 89% and a precision of 79% without data augmentation, which improved to 92% accuracy and 81% precision in ResNet50V2after augmentation.

### Herlev dataset

The results of binary classification on the Herlev dataset are shown in Table 2. For segmentation, the improved U-Net slightly increased accuracy from 88.34% to 88.39%, and RES_DCGAN before segmentation further improved it to 88.95%. For classification, the Normal pipeline generally outperformed ROI-based methods. In the ensemble method, accuracies were 91% and 87% without RES_DCGAN, and 89% and 86% with RES_DCGAN applied after segmentation for the Normal and ROI methods, respectively. Adding RES_DCGAN before segmentation did not further improve ensemble performance. This suggests that, although the Herlev dataset is diverse, the ensemble method already generalizes well, and the RES_DCGAN augmentation provides limited additional benefit for this dataset.

**Table 2:** The result of the study using the Herlev dataset in binary classification.

Methods/pipelines	Segmentation					Classification											
						ResNet50V2				Voting				Ensemble			
	Acc	Pre	Rec	Dice	IoU	Acc	Pre	Rec	F1	Acc	Pre	Rec	F1	Acc	Pre	Rec	F1
Standard U-Net	88.34	91.5	92.27	91.77	90.41	90	91	90	90	89	90	89	88	87	89	87	86
Normal	88.39	92.16	91.56	89.49	91.93	90	91	90	90	86	88	86	85	91	91	91	90
Nor_RES_DCGAN						88	90	88	87	88	88	88	88	89	89	89	89
ROI						82	81	82	80	86	87	86	85	87	88	87	86
ROI_RES_DCGAN						80	79	80	80	50	69	50	51	86	86	86	85
RES_DCGAN_Nor	**88.95**	92.4	92.56	90.33	**92.31**	88	89	88	86	86	86	86	86	**91**	**91**	**91**	**91**
RES_DCGAN_ROI						82	85	82	79	85	87	85	84	87	88	87	86

### SIPaKMeD dataset

The results for the multi-class classification on the SIPaKMeD dataset are summarized in Table 3.

**Table 3:** The result of the study using the SIPaKMeD dataset in multi-class.

Methods/pipelines	Segmentation					Classification											
						ResNet50V2				Voting				Ensemble			
	Acc	Pre	Rec	Dice	IoU	Acc	Pre	Rec	F1	Acc	Pre	Rec	F1	Acc	Pre	Rec	F1
Standard U-Net	94.28	96.27	92.58	93.68	95.37	91	91	91	91	92	92	92	92	94	94	94	94
Normal	98.01	98.23	97.91	97.13	98.37	92	92	92	92	92	93	92	92	**95**	**95**	**95**	**95**
Nor_RES_DCGAN						89	89	89	89	94	94	94	94	94	95	94	94
ROI						83	83	83	83	84	86	84	83	86	86	86	86
ROI_RES_DCGAN						85	86	85	85	76	85	76	77	90	90	90	90
RES_DCGAN_Nor						92	92	92	92	93	94	93	93	94	94	94	94
RES_DCGAN_ROI	**98.3**	98.44	98.28	97.38	**98.59**	81	82	81	80	88	88	88	88	90	90	90	90

For segmentation, the improved U-Net achieves 98.01% accuracy, outperforming the standard U-Net (94.28%). Adding RES_DCGAN before segmentation slightly increased accuracy to 98.3%. In classification, the Normal pipeline consistently outperforms ROI-based methods, achieving up to 95% accuracy with the ensemble method. The results of the binary classification of the SIPaKMeD dataset are shown in Table 4.

**Table 4:** The result of the study using the SIPaKMeD dataset in binary classification.

Methods/pipelines	Segmentation					Classification											
						ResNet50V2				Voting				Ensemble			
	Acc	Pre	Rec	Dice	IoU	Acc	Pre	Rec	F1	Acc	Pre	Rec	F1	Acc	Pre	Rec	F1
Standard U-Net	94.28	96.27	92.58	93.68	95.37	97	97	97	97	97	97	97	97	98	98	98	98
Normal	98.01	98.23	97.91	97.13	98.37	96	96	96	96	97	97	97	97	**99**	**99**	**99**	**99**
Nor_RES_DCGAN						97	97	97	97	97	97	97	97	**99**	**99**	**99**	**99**
ROI						89	89	89	89	95	95	95	95	96	96	96	96
ROI_RES_DCGAN						90	90	90	90	93	93	93	93	95	95	95	95
RES_DCGAN_Nor						96	96	96	96	98	98	98	98	98	98	98	98
RES_DCGAN_ROI	**98.3**	98.44	98.28	97.38	**98.59**	90	90	90	90	95	95	95	95	96	96	96	96

For binary classification, the ensemble method achieves the highest performance, scoring 99% accuracy both with and without RES_DCGAN in the Normal pipeline. ROI-based methods achieve slightly lower accuracy (95–96%). Adding RES_DCGAN before segmentation did not significantly affect ensemble performance, which remained at 98% accuracy. The summary of the optimal result from the different datasets is depicted in Table 5.

**Table 5:** Optimal segmentation and classification pipelines across datasets.

Dataset	Best segmentation method	Seg. Acc/IoU	Best classification method	Class. acc/F1
Pomeranian	RES_DCGAN_ROI	99.53%/99.43%	Normal	96%/96%
Herlev	RES_DCGAN_Nor	88.95%/92.31%	RES_DCGAN_Nor	91%/91%
SIPaKMeD (multi-class)	RES_DCGAN_ROI	98.30%/98.59%	Normal	95%/95%
SIPaKMeD (binary)			Normal/Nor_RES_DCGAN	99%/99%

**Fig. 1:**
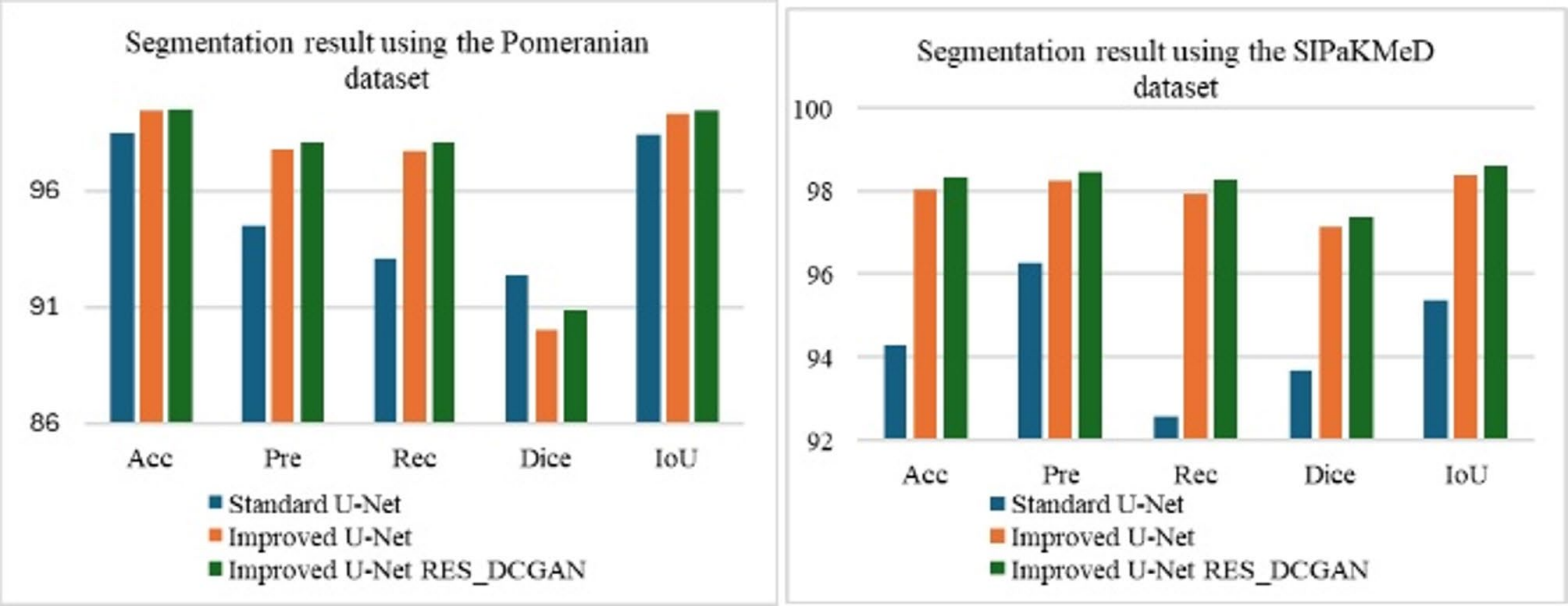
Comparison of segmentation output on the Pomeranian and SIPaKMeD dataset.

As shown in Table 5, the RES_DCGAN_ROI pipeline consistently achieved the best segmentation results across all datasets, indicating its effectiveness in improving region-level feature representation. For classification, the Normal pipeline was superior on the Pomeranian and SIPaKMeD datasets, while RES_DCGAN_Nor performed best on the Herlev dataset. In binary classification with SIPaKMeD, both the Normal and Nor_RES_DCGAN pipelines reached the highest accuracy and F1-score (99%). These findings suggest that while RES_DCGAN_ROI is good for a segmentation strategy, the optimal classification pipeline may vary depending on the dataset. Sample segmentation of the Pomeranian and SIPaKMeD dataset is shown in Fig. 1.

**Fig. 2:**
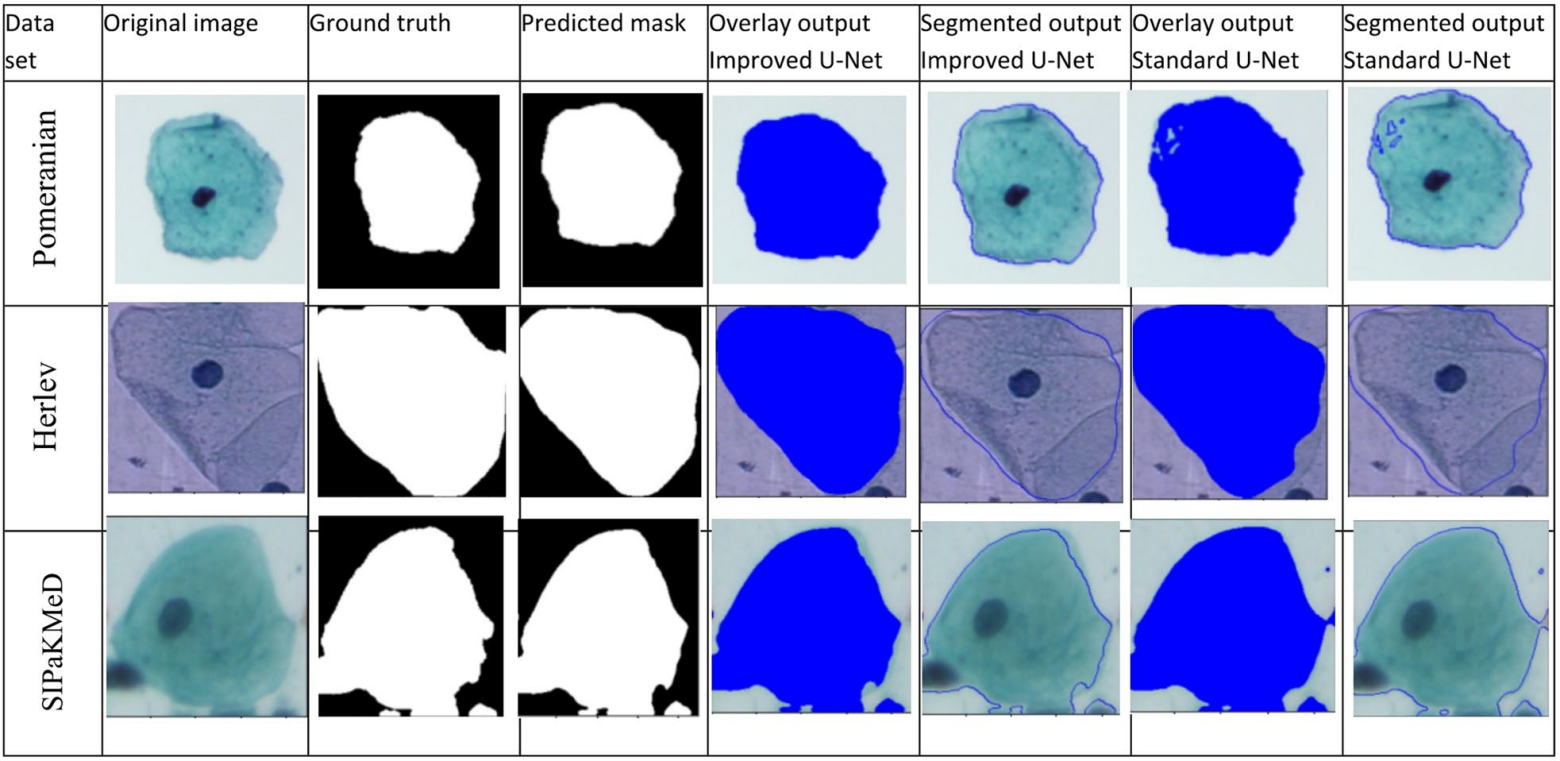
Segmentation output for the study.

Sample images from all datasets, illustrating the segmentation results using the improved U-Net architecture, are presented in Fig. 2. In the predicted masks, whole cells are highlighted in white, and the background appears in black. The first column contains the original image, the second column the ground truth mask, the third column the predicted mask, the fourth column the overlay of the predicted mask, and the fifth column the segmented output based on the improved U-Net prediction. The sixth and seventh columns present the overlay and segmented output obtained using the standard U-Net, respectively.

**Fig. 3:**
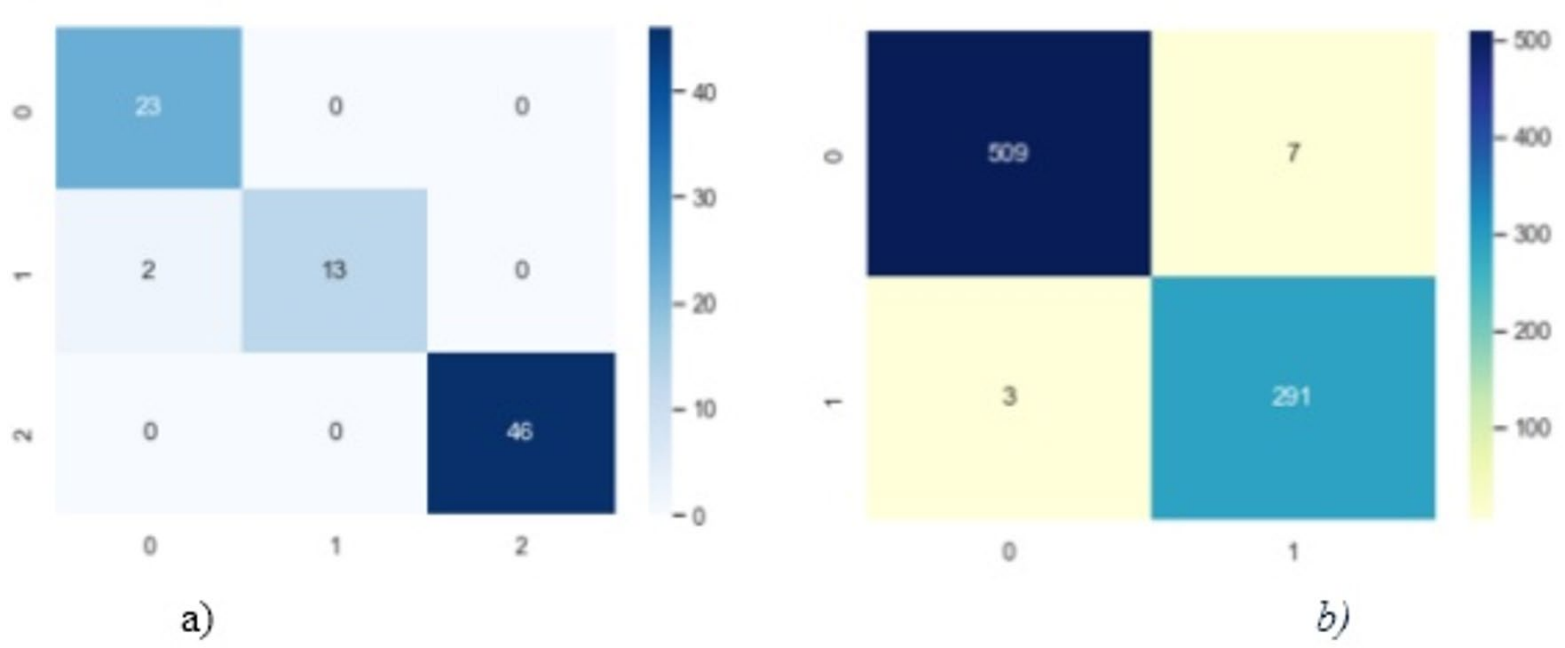
Sample confusion matrix: (**a**) Pomeranian, (**b**) SIPaKMeD binary classification.

**Fig. 4:**
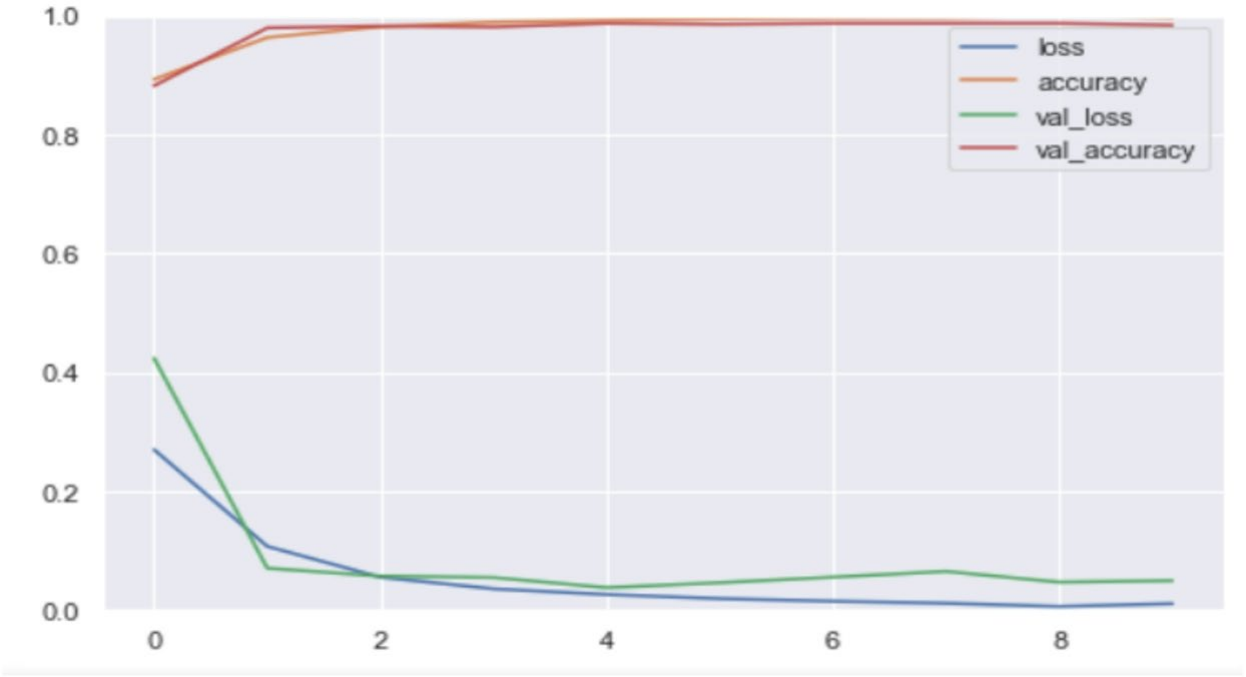
Training versus validation accuracy and loss.

As illustrated in Fig. 2, the Pomeranian dataset reveals some discrepancies, with black areas in the whole cell, not present in the actual mask, and some lines circled in blue in the segmented result. These issues are absent from the improved U-Net architecture. Similarly, in other datasets, the improved U-Net provides segmentation results that more closely match the actual images compared to the standard U-Net. This indicates that our method is more accurate for whole-cell segmentation. Figure 3 also illustrates the confusion matrix for the proposed method, and Fig. 4 shows the proposed training versus validation accuracy and loss for the binary classification of the SIPaKMeD dataset.

In Fig. 3, only two samples are misclassified, and 82 samples are correctly classified in the Pomeranian dataset. In the SIPaKMeD dataset, 10 samples are misclassified in binary classification, while 800 samples are correctly classified in these respective tasks. As can be shown in Fig. 4, there was an early stopping during model training. The training and validation accuracies are almost identical, and the model stops at the eighth epoch, achieving an accuracy of 99%. The validation loss decreases from 0.4 to 0.05, while the training loss decreases from 0.25 to nearly zero. Both the training and validation graphs demonstrate the effectiveness of the model in achieving high accuracy and low loss, which is crucial for cervical cell classification.

## Discussions

Now it is time to summarize the key findings of our study and answer the research questions. RQ1: Can segmentation improve classification performance? Yes, segmentation significantly improves classification by isolating meaningful regions of interest in cervical cell images. In the Herlev dataset, accuracy increased from 87 to 91%, and in SIPaKMeD multi-class and binary classification, accuracy improved from 94 to 95% and from 98 to 99%, respectively. This demonstrates that segmentation is particularly beneficial, even without RES_DCGAN augmentation. RQ2: Does RES_DCGAN improve the robustness and generalizability of the segmentation model? Yes, applying RES_DCGAN before segmentation generally increases segmentation performance, although the improvement is slight for the more diverse Herlev dataset. RQ3: Which classification strategies achieve the best performance? For the Pomeranian and SIPaKMeD datasets, the Normal pipeline consistently achieved the highest classification performance, whereas for the Herlev dataset, RES_DCGAN_Nor was superior. In summary, segmentation enhances classification, RES_DCGAN strengthens segmentation robustness, and the optimal classification strategy varies across datasets.

This paper introduces a segmentation approach for Pap smear images using an improved U-Net architecture. This architecture incorporates a dilation layer and group normalization to increase the receptive field and capture more contextual information for accurate pixel-wise predictions. Group normalization stabilizes and speeds up the training process by normalizing a group of batches. We proposed six different pipelines for the segmentation and classification of Pap smear images to detect cervical cancer: normal method, Nor_RES_DCGAN, ROI, ROI_RES_DCGAN, RES_DCGAN, Nor, and RES_DCGAN_ROI, as discussed earlier. For segmentation, we conducted two types of experiments: using the improved U-Net with the real dataset for the first four pipelines and adding RES_DCGAN before segmentation for the last two pipelines. To evaluate the performance of the proposed model, we used the Pomeranian, Herlev, and SIPaKMeD datasets. The improved U-Net outperforms the standard U-Net, with RES_DCGAN added before segmentation, slightly improving performance. The proposed segmentation model achieved accuracies of 99.53%, 88.95%, and 98.3% for the Pomeranian, Herlev, and SIPaKMeD datasets, respectively.

For classification, we implemented ResNet50V2 and an ensemble of different pretrained models to enhance performance. These ensembles include majority voting across pretrained models and feature concatenation from these models. The classification pipelines involve the normal method, ROI, and RES_DCGAN added before and after segmentation of the normal method and ROI as inputs. The normal method generally yields better results, followed by the scenarios where RES_DCGAN is added before and after segmentation. The model achieved accuracies of 96% and 91% on the Pomeranian and Herlev, and 99% and 95% for binary and multi-class classification on the SIPaKMeD datasets, respectively. Table 6 compares the performance of various segmentation methods with our proposed method.

The approach used by Desiani et al.^10^ applied a CNN-based U-Net architecture to segment images for an accurate diagnosis of cervical cancer. Our approach enhances this by incorporating dilation in the encoder and group normalization in both the encoder and decoder of the U-Net architecture, using the same dataset. Their model achieved an accuracy of 77%, while our method achieved 88.95% accuracy. Alisha and Vinitha^18^ achieved 91.8% precision and 92.1% recall, focusing on the automated segmentation of cervical cell nuclei and integrating it with other deep- learning models to enhance efficiency. Our study surpassed these results, achieving a precision of 92.4% and a recall of 92.56%. Gang Li et al.^19^ introduced a module with global dependency and local attention (GDLA) mechanisms to segment cervical cells, leveraging channel attention, global dependency, and spatial attention. This module captures information on the positions of the cell, i.e., nuclei and cytoplasm, and achieves a precision of 80.5% and a recall of 78.7%. Our improved U-Net architecture outperforms this, achieving a recall of 92.56%.

**Table 6:** Comparison of the results of our study with previous studies.

Refs.	Dataset	Class	Segmentation						Classification				
			Methods	Acc	Pre	Rec	Dice	IoU	Methods	Acc	Pre	Rec	F1
^10^	Herlev	2	U-Net	77	–	72	–	–	–	–	–	–	–
^18^	Herlev	2	EfficientDet	–	91.8	92.1	–	83.6	**–**	**–**	–	**–**	**–**
^19^	Herlev	2	GDLA Unet	–	80.5	78.7	–	–	**–**	**–**	**–**	**–**	**–**
^20^	Herlev	2	DIFF	–	–	–	–	–		94.55	94.1	91.6	92.7
^21^	Herlev	2	Hierarchical	–	–	–	90	–	HMLS	89	–	–	–
^11^	Herlev	2	MaskRCNN	–	92	91	–	–	VGG Net	–	–	96	–
^12^	Herlev	2	Pyramid scene	96	96	96	–	74	Ensemble	99.7	–	–	–
^22^	Herlev	2	Weekly supervise	–	–	–	62.05	61.89	VGG16 adapted 128	94	94	94	94
^23^	SIPaKMeD	5	–	–	–	–	–	–		91.5	–	–	–
^24^	SIPaKMeD	5	–	–	–	–	–	–		92	–	–	–
^25^	SIPaKMeD	5	–	–	–	–	–	–	MLP	91.72	91	91.6	91.7
^13^	SIPaKMeD	2	–	–	–	–	–	–	ResNet50	93.7	–	–	–
^26^	SIPaKMeD	2	–	–	–	–	–	–	HDFF	99.8	–	–	–
^27^	SIPaKMeD	2	SE-DeepLabV3+	96.09	97.04	96.13	96.00	96.52	Ensemble	98	98	98	98
^28^	SIPaKMeD	2	SPP-SegNet	94.15	93.87	94.94	–	95.08	SE-DenseNet201	99	99	99	99
Ours	Herlev	2	Improved U-Net	**88.95**	92.4	92.56	90.33	92.31	Ensemble	91	91	91	90
Ours	SIPaKMeD	5	Improved U-Net	**98.3**	98.44	98.28	97.38	98.59	Ensemble	95	95	95	95
Ours	SIPaKMeD	2							Ensemble	99	99	99	99
Ours	Pomeranian	3	Improved U-Net	**99.53**	98.09	98.09	90.88	99.43	Ensemble	96	96	96	96

Fang et al.^20^ proposed a deep learning approach for cervical cell classification using the Herlev dataset. They developed a deep integrated feature fusion (DIFF) block to combine local and global features from both a CNN branch and a transformer branch. There was a 1 × 1 convolution in the DIFF block to enhance channel interactions in the concatenated feature maps, producing richer representations than simple stacking. Additionally, skip connections retain the original features, minimizing information loss in deeper layers. This interactive integration improves communication between local and global features. Their model achieved an accuracy of 94.55%, which is higher than that of our study. Braga et al.^21^ proposed a hierarchical median narrow band level set approach (HMLS) for segmenting nuclei in cervical cells. Braga used a multi-scale analysis algorithm to estimate the number of clusters in each cell-containing image region, which then serves as input for a narrow-band level set al.gorithm. Their model achieved a 90% Dice coefficient for segmentation and 89% classification accuracy. Our results show a slightly higher Dice coefficient of 90.33% and an accuracy of 91%.

Kurnianingsih et al.^11^ and Sabeena and Gopakumar^12^ used the Herlev dataset for segmentation and classification. Kurnianingsih et al. used Mask R-CNN with ResNet10 for automatic segmentation, achieving 92% precision and 91% recall. They then used VGG Net for binary classification, achieving 96% recall. Our study achieved a recall of 92.56% in segmentation. Sabeena and Gopakumar used a pyramid scene parsing model for segmentation, achieving 96% accuracy and 74% IoU. Although its accuracy is higher, our study has a better IoU of 92.31%, indicating more overlap between the predicted mask and the ground truth. They achieved 99.7% accuracy in classification using an ensemble of Naïve Bayes, MLP, random forest, J48, and support vector machine classifiers. Sritharan et al.^22^ achieved 62.05% Dice and 61.89% IoU with weakly supervised segmentation on Herlev, which is far lower than our improved U-Net (Dice 90.33%, IoU 92.31%), underscoring the superiority of our method. However, the classification result is superior, which is 94%.

In addition to the studies summarized in Table 6, several recent works provide broader perspectives on cervical cell analysis. Sarhangi et al.^29^ reviewed deep learning approaches across cytology and colposcopy, highlighting segmentation and classification advances but noting limitations in dataset diversity and the integration of both tasks within a single framework. Gangrade et al.^30^ proposed an ensemble model for SIPaKMeD classification, achieving 94% accuracy, yet without segmentation or augmentation for robustness. Fang et al.^31^ systematically reviewed cervical cell image analysis methods and observed that CNN- and transformer-based classifiers are common, while segmentation is largely dominated by standard U-Net/FCN designs with limited generalizability across datasets. Compared to these studies, our approach (i) unifies segmentation and classification in a single pipeline, demonstrating that segmentation consistently improves classification accuracy across multiple datasets, and (ii) introduces RES_DCGAN augmentation, enhancing segmentation robustness and dataset generalization. Quantitatively, our method achieves higher segmentation overlap (Dice up to 97.13%, IoU up to 98.37%) while maintaining competitive classification accuracy (up to 99%), outperforming ensemble-only classification^30^ and weakly supervised segmentation^22^. These results highlight both the relevance and the novelty of our framework in the context of currently available methods.

On the other hand, for the SIPaKMeD dataset, previous work has focused solely on classification. In contrast, our study includes both segmentation and classification by creating a corresponding mask for segmentation. Song et al.^23^ addressed the challenge of variations in the appearance and shape of cervical cells, which can lead to under-representative training data. Their approach was designed to improve classifier generalizability by focusing on worst-case data with larger gradient norms, using worst-case enhancement to improve learning from under-represented samples. They achieved an accuracy of 91.5%. Vatsala and Prabhnoor^24^ performed an effective cervical cell classification using CNN and achieved an accuracy of 92% in the five-class classification. However, our study achieved an accuracy of 95%. Liu et al.^25^ proposed a deep learning system for cervical cell classification, where a CNN extracts a feature and an MLP combines local and global features. Their model achieved an accuracy of 91.72%, a precision of 91%, a recall of 91.6%, and an F1 score of 91.7% in multi-class classification. Our study outperforms this by achieving 95% in all performance metrics.

In binary classification, Tripathi et al.^13^ and Rahaman et al.^26^ conducted studies using the SIPaKMeD dataset. Tripathi et al.^13^ classified cervical cells in different stages of cancer progression using deep learning models, eliminating the need for manual feature extraction. They employed ResNet50, ResNet152, VGG16, and VGG19, achieving accuracy of 93.7%, 94.89%, 92.85%, and 94.3%, respectively. In comparison, our method achieves 99% accuracy in all performance metrics for the two-class problem. Rahaman et al.^26^ used four deep learning models, integrating their features with a hybrid deep feature fusion (HDFF) approach to classify cervical cells. The transfer learning algorithms applied in this technique include VGG16, VGG19, Xception, and ResNet50. Their proposed methods achieved an accuracy of 99.8%, whereas our study achieved 99% accuracy using the ensemble method. In^27^, SE block was integrated into the ASPP module of the DeepLabV3+, the model scored an accuracy of 96.09%, while in^28^ the SPP block was added at the bottleneck of the SegNet architecture to segment the image, and the model achieves an accuracy of 94.15% on the SIPaKMeD dataset, which is lower than our result.

Overall, our study addresses key limitations of prior works by unifying segmentation and classification within a single framework, while introducing a novel application of RES_DCGAN both before and after segmentation. Unlike studies such as Sritharan et al.^22^, which rely on weakly supervised segmentation with relatively low Dice and IoU scores, or others that focus only on classification^23–25^, our pipeline achieves high segmentation accuracy (Dice up to 97.38%, IoU up to 98.59%) alongside competitive classification results (up to 99% in binary and 95% in multi-class tasks) on the SIPaKMeD dataset. The integration of RES_DCGAN improves dataset diversity and model generalizability, particularly in cases with limited training data. Thus, our framework not only delivers superior segmentation but also ensures consistent classification performance across diverse datasets.

Despite the promising performance of the proposed segmentation and classification framework, several limitations remain. The model shows slightly lower accuracy on the Herlev dataset, likely due to higher variability in cell morphology. Additionally, for the Pomeranian and SIPaKMeD datasets, the segmentation masks were manually generated and not validated by expert cytologists, which may introduce minor inaccuracies. The stochastic nature of deep learning models can cause minor variations across runs. Furthermore, the framework was tested only on single-cell images, so its performance on multi-cell or clinical slides is unverified. While RES_DCGAN-based augmentation improves segmentation slightly, the benefits vary across datasets. In this study, model validation was performed on the same individuals used for training, which could limit generalizability. Future work will focus on testing the framework on larger and more diverse multi-cell datasets, incorporating expert-validated masks, and evaluating performance across different clinical populations with external validation.

## Methods

This section presents the data used and their preprocessing technique, the method used, the tools, and the experimental settings for the study.

### Data acquisition and preprocessing

The data used for this study included the private Pomeranian dataset from the Pomeranian Medical University in Szczecin, Poland, as well as the publicly available SIPaKMeD and Herlev single-cell datasets. The first Pap smear images used in the study were collected in BMP format as RGB color images, with a resolution of 1130 × 1130 pixels. A total of 419 cervical cell images were used, consisting of 124 high-grade squamous intraepithelial lesion (HSIL) images, 61 low-grade squamous intraepithelial lesion (LSIL) images, and 234 normal squamous intraepithelial lesion images^32^.

**Fig. 5:**
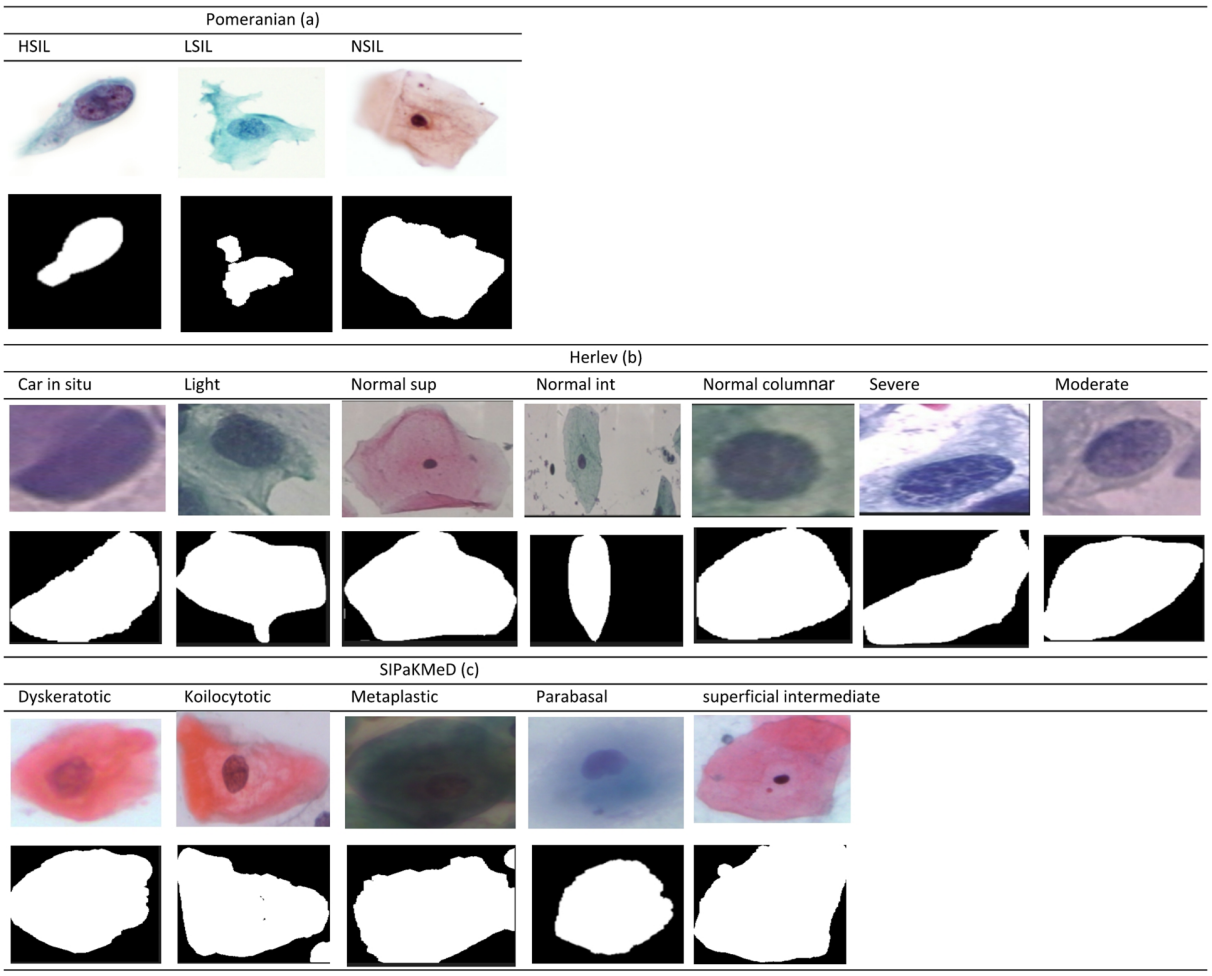
Sample images with their corresponding mask: (**a**) Pomeranian, (**b**) Herlev, (**c**) SIPaKMeD.

The second dataset, the publicly available Herlev dataset^33^, contains 917 Pap smear images categorized into seven distinct classes representing different types of cervical cells. This dataset was collected at Herlev University Medical Center using a microscope and digital camera, and it was established through evaluations by doctors and two cytotechnicians^6^. The dataset includes ground truth labels used for segmentation tasks. The classes are squamous cell carcinoma in situ, severe squamous non-keratinizing dysplasia, moderate squamous non-keratinizing dysplasia, mild squamous non-keratinizing dysplasia, normal columnar epithelial, intermediate squamous epithelial, and superficial squamous epithelial cells. The first four are considered abnormal, while the last three are considered normal for binary classification. The third dataset, SIPaKMeD^34^, consists of 4049 cervical cell images manually cropped from 966 cluster cell images of Pap smear slides. These images are divided into five categories: superficial, intermediate, parabasal, metaplastic, koilocytotic, and dyskeratotic^35^. Figure 5 shows some example images from the Pomeranian, Herlev, and SIPaKMeD datasets. The details of the datasets are described in Table 7.

**Table 7:** Details of the dataset used in the study.

Pomeranian			SIPaKMeD				Herlev			
Cervical cell images	No. of images	Generated images	Cervical cell images	No. of images	Generated images	Category	Cervical cell images	No. of images	Generated images	Category
HSIL	124	901	Dyskeratotic	813	787	Abnormal	Car in situ	150	895	Abnormal
LSIL	61	951	Koilocytotic	825	775		Sever	197	862	
NSIL	234	812	Metaplastic	793	807		Moderate	146	897	
							Mild squamous	182	872	
			Parabasal	787	813	Normal	Normal-columnar	98	931	Normal
			Superficial-intermediate	831	769		Intermediate	70	951	
							Superficial	74	949	
Image size (pixel)	1130 × 1130		Different				Different			
Total	**419**	2664	**4049**		3951		**917**		6357	

The publicly available images have a low contrast between the cells and the background, and some are blurred. Therefore, we pre-process these images by adjusting their brightness and contrast. During preprocessing, image sizes were standardized to 224 × 224 pixels to ensure consistent input for the model and normalized to the range [0,1] to facilitate faster learning. For the Pomeranian and SIPaKMeD datasets, binary masks were manually created using OpenCV to highlight the entire cell, including cytoplasm and nucleus. This approach, however, has a limitation, as the masks were not validated by experts, which may introduce minor inaccuracies in segmentation evaluation. In contrast, the Herlev dataset initially used color masks, which we converted to binary masks. This conversion helps to isolate the region of interest (ROI) effectively and simplifies the separation of relevant areas from the background when multiplying with the original image.

### Proposed model

This section presents the proposed pipelines for segmenting and classifying Pap smear images to detect cervical cancer. The first phase is segmentation, which divides the image into different parts and identifies the region of interest (ROI) to detect cervical cancer. This is followed by a classification phase that categorizes cervical cancer types into predefined categories. Due to the limited amount of data, we used residual deep convolutional generative adversarial network (RES_DCGAN) data augmentation techniques to generate artificial data and improve the model’s performance and generalizability.

### Segmentation

In the first phase, we used a segmentation technique based on the U-Net architecture^36^, which has demonstrated state-of-the-art performance in various medical imaging modalities. The U-Net architecture, introduced by Ronneberger et al. in 2015 for the segmentation of biomedical images in the ISBI2015 segmentation challenge, gained attention due to its innovative approach of reusing feature maps from the encoder in the network’s decoder^37^. This fully convolutional design proved to be well-suited for the semantic segmentation task. In this study, we proposed a U-Net-based segmentation architecture to segment the entire cell from the background in Pap smear images. In the encoder, features were extracted while the spatial dimensions were progressively reduced and their depth increased. Each convolutional block included two convolutional layers followed by group normalization, an activation function, a MaxPooling operation, and dropout in the final block.

In the convolutional layers, we added a dilation rate to increase the receptive field without increasing the number of parameters or losing spatial resolution. This allows the model to capture more contextual information, which is crucial for accurate pixel-wise predictions. It also enables the model to detect larger and more complex features at different scales, enhancing its ability to distinguish between various regions or objects in an image. Group Normalization, which normalizes over groups of channels rather than individual channels, provides greater stability when the batch size is small. This helps stabilize and speed up the training process while capturing variability within groups of features, thus improving the model’s ability to learn and generalize from the data.

The decoder, on the other hand, decodes the encoded data using information from the concatenation, and the spatial dimensions are expanded to recover image resolution and preserve location information. It comprises two convolutional blocks followed by a group normalization, activation function, and dropout in the last block.

### Data augmentation

We implemented a data augmentation technique to increase the size of the training dataset using RES_DCGAN, a variant of the Deep Convolutional Generative Adversarial Network (DCGAN) with residual blocks added to the generator^38^. This modification improves feature learning and training stability. The RES_DCGAN model consists of a generator, which creates synthetic images, and a discriminator, which distinguishes real from generated images. The generator maps a random noise vector (reshaped to 6 × 6) through four up-sampling layers to produce images of size 96 × 96 × 3, with decreasing filter sizes from 512 to 64. The discriminator receives real and generated images of the same size and uses five convolutional layers with increasing filters (32 to 512) to distinguish real from synthetic images. The performance of the generated images was evaluated using the Fréchet Inception Distance (FID), which measures their similarity to real images. The proposed RES_DCGAN has the smallest FID value compared to the standard DCGAN augmentation.

This augmentation was applied both before segmentation and after the segmentation (i.e., classification) phase. The real images were divided into training (64%), validation (16%), and testing (20%) sets, while the generated images were divided into training (80%) and validation (20%) sets. When RES_DCGAN was applied before segmentation, the generated images were mixed with the real images (i.e., generated training with real training and generated validation with real validation) to train the segmentation model. The real test set, which was not used to generate synthetic images, was then used to evaluate segmentation performance. When RES_DCGAN was applied after segmentation, it served as input for the classification task, with generated data mixed with real training and validation data, while the previously unused test set was employed for classification evaluation. This approach increased the size of the training data and improved the performance and generalizability of the models. By adding RES_DCGAN before segmentation, we aimed to assess whether augmenting the dataset at the segmentation stage enhances the quality of the segmented features, thereby improving downstream classification. Conversely, applying RES_DCGAN after segmentation allows us to investigate whether augmenting only the inputs to the classification task is sufficient for improving performance. This dual strategy enables a comparative analysis of the impact of augmentation at different stages of the pipeline, helping us identify the optimal approach for robust cervical cell classification.

### Classification

**Fig. 6:**
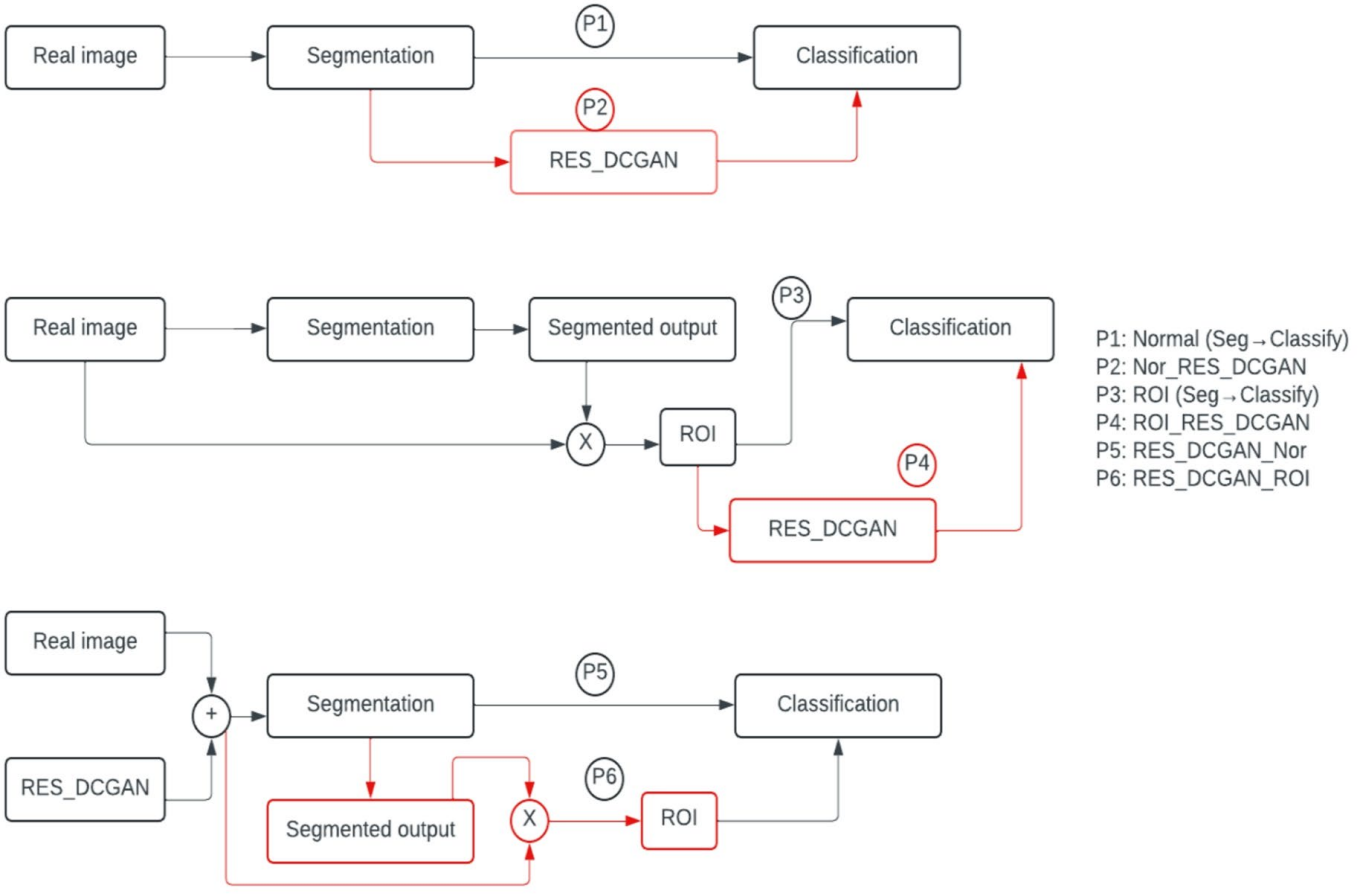
The proposed pipelines for the study.

**Fig. 7:**
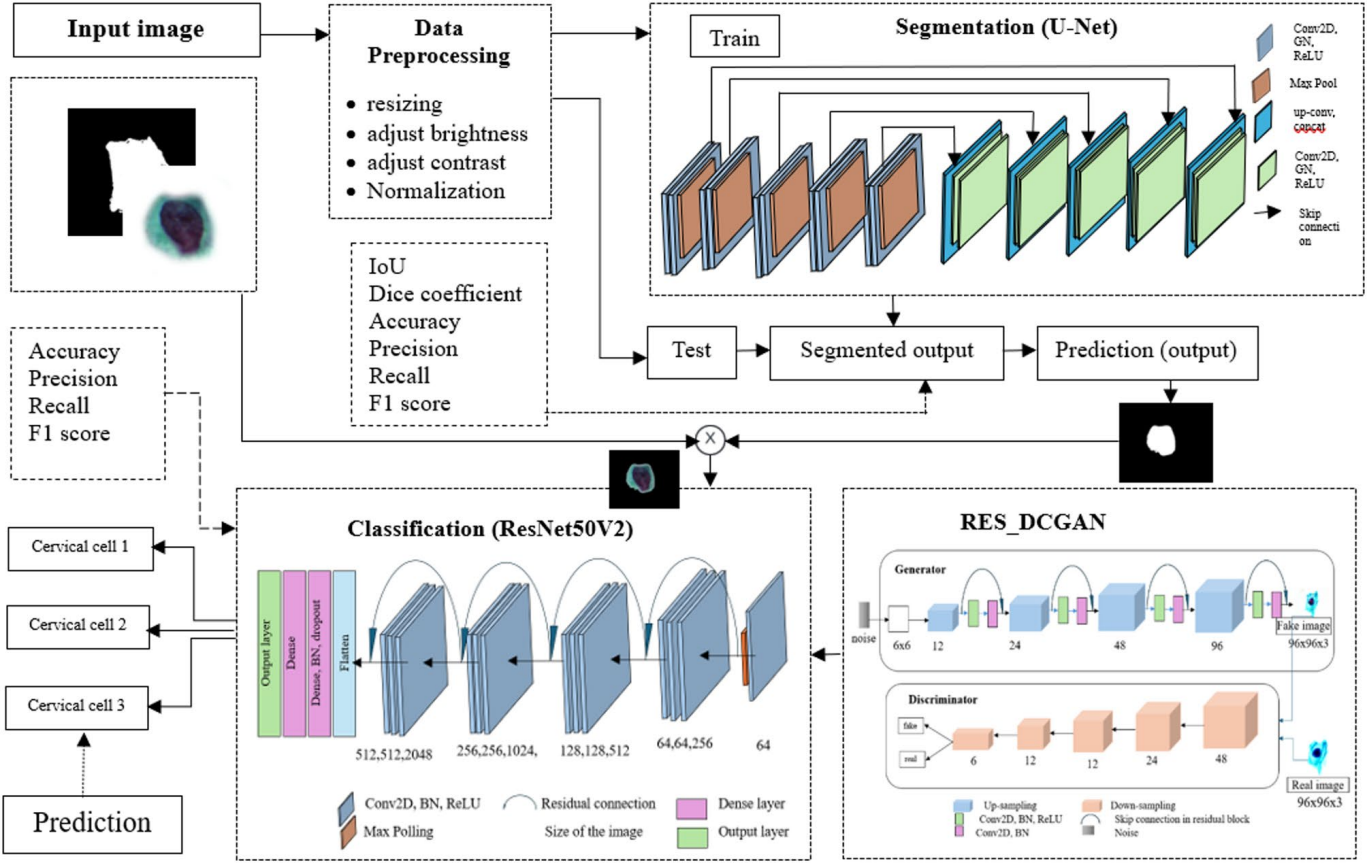
The proposed model for the detection of cervical cancer.

The second phase is the classification task, which categorizes cervical cells into their predefined classes. We employ a ResNet50V2 and an ensemble of different pretrained networks as the basis for our deep-learning model in this classification stage. ResNet50V2 is part of the ResNet family that helps in solving the problem of vanishing gradients in deep networks, which allows deeper architectures to be effectively trained. It is one of the prominent deep learning models that introduced the idea of residual learning^39^. In the ResNet architecture, residual connections are used to enhance the deep network training process by allowing information to pass through multiple layers more effectively. The proposed pipelines are depicted in Fig. 6, and the model used in the study is depicted in Fig. 7.

This study uses six different pipelines for the detection of cervical cancer. The first pipeline involves performing segmentation followed by classification. The second pipeline is like the first, but includes RES_DCGAN after segmentation, which is then used for classification. The third pipeline performs segmentation and multiplies the segmented mask by the original images, using the resulting product, the region of interest (ROI), as input for the classification task. The fourth pipeline is like the third but incorporates RES_DCGAN into the ROI after segmentation, which is then used for classification. In the fifth pipeline, RES_DCGAN is applied before segmentation, followed by the classification task. Finally, the sixth pipeline is like the fifth but uses the segmented output multiplied by the original image as input for classification. Implementing these six different pipelines with advanced techniques, such as automatic cell analysis, plays a vital role in early disease identification and improving treatment outcomes.

The ensemble method involves a majority voting technique implemented to combine predictions from multiple deep-learning models. We trained seven different pretrained models (ResNet50V2, VGG19, VGG16, Xception, DenseNet121, EfficientNetB2, and MobileNetV2) on a dataset, and each model makes predictions on the test data. These models were selected because they are well-established CNN architectures with complementary strengths, allowing the ensemble to leverage their diverse feature representations and improve overall classification accuracy and robustness across datasets. These predictions are then aggregated using majority voting, where the final predicted class for each test sample is determined by the most frequent prediction across all models. This approach aims to leverage the strengths of each model to improve overall classification accuracy. In another ensemble method, we combined features from eight different pretrained CNN architectures, those used in the majority voting ensemble, plus InceptionV3. Each model is used to extract features from the input images, and these features are then flattened and concatenated into a single feature vector. The combined features are passed through fully connected layers with Batch Normalization and Dropout to reduce overfitting and improve training stability. The final layer outputs class probabilities for different classes.

To verify the flexibility of our method, we applied the same segmentation and classification pipeline to multiple datasets with different numbers of classes. Only the final output layer of the classification model was adjusted to match the specific number of classes, demonstrating that our approach can generalize across datasets.

### Experimental setting

The research experiment was carried out on a server running Windows 10, an AMD Ryzen 9 5950 × 16-core processor, 128GB of RAM, and an NVIDIA GeForce RTX 3090 GPU. We use Python and TensorFlow with the Keras framework for implementation. For the segmentation task, we employed an improved U-Net architecture with dilated convolutions and group normalization. The model was trained using a batch size of 18 for 100 epochs with the Adam optimizer (learning rate 0.003) and Jaccard distance as the loss function. Spatial dropout (0.5) was applied to reduce overfitting. For classification, we implemented an ensemble of eight pretrained CNN models (ResNet50V2, VGG19, Xception, InceptionV3, VGG16, EfficientNetB2, MobileNetV2, DenseNet121) with frozen weights. Features were extracted, flattened, and concatenated before passing through fully connected layers with 256 and 64 neurons, Batch Normalization, and Dropout (0.5) before the final softmax layer. The ensemble was trained with the Adam optimizer, categorical cross-entropy loss, batch size of 32, and 100 epochs. Both segmentation and classification tasks utilized the augmented datasets generated via RES_DCGAN. Data augmentation required different times depending on the dataset size: approximately 1 h and 45 min per class for Pomeranian, 2 h and 30 min for SIPaKMeD, and 28 min for Herlev. With early stopping, the average training time was about 5–9 min for segmentation models and 8–15 min for the ensemble classifier. The complexity analysis for the segmentation and classification techniques is depicted in Table 8. The codes for this research are available from the corresponding author.

As can be seen in Table 8, total, trainable, and non-trainable parameters are reported to show computational cost. Segmentation models are smaller but fully trainable. The classification ensemble has significantly higher total parameters due to combining eight CNN backbones; however, more than half of these are frozen pretrained weights.

**Table 8:** Complexity analysis of the study.

Pipeline/model	Total parameters	Trainable parameters	Non-trainable parameters	Avg. epoch time	Remarks
Standard U-Net	31,466,753	31,454,721	12,032	~ 26–46 s	Baseline segmentation model
Improved U-Net	31,454,721	31,454,721	0	~ 35–51 s	Improved UNet, no generative augmentation
Improved U-Net with RES-DCGAN	31,454,721	31,454,721	0	~ 35–51 s	Improved UNet with GAN-based augmentation
Ensemble of 8 pretrained CNNs	241,946,310	123,913,477	118,032,833	~ 25–48 s	Features extracted from frozen pretrained CNNs, concatenated, then Dense layers

## Conclusions

This study proposed a framework for cervical cell image segmentation and classification using an improved U-Net architecture. The approach was evaluated on the Pomeranian, Herlev, and SIPaKMeD datasets across six pipelines. For segmentation, the RES_DCGAN_Nor pipeline, which includes data augmentation prior to segmentation, produced slight improvements over the normal pipeline. For classification, the Normal pipeline generally achieved the highest accuracy, though other pipelines with RES_DCGAN augmentation also performed competitively in the ensemble method. Overall, the results indicate that the proposed methods can enhance segmentation and classification performance on single-cell Pap smear images, although improvements were modest and dataset-specific. Future work will investigate extensions to multi-cell images, the use of deeper networks, and advanced preprocessing techniques to further refine performance while addressing dataset variability and mask quality limitations.

## Data Availability

The SIPaKMeD and Herlev datasets used in this study are publicly available at the following links: SIPaKMeD: [https://www.cs.uoi.gr/~marina/sipakmed.html] Herlev: [https://mde-lab.aegean.gr/index.php/downloads/]. The Pomeranian dataset was provided by the Pomeranian Medical University in Szczecin, Poland, under a restricted data-use agreement. As we are not the owners of this dataset, it cannot be made publicly available. The dataset is available from the corresponding author upon reasonable request 10.5281/zenodo.17642009 and 10.5281/zenodo.17620636.

## Code availability

All custom code developed for this study, including the U-Net segmentation model, ResNet50V2 classifier, ensemble models, RES-DCGAN augmentation, and the six integrated pipelines, is publicly available on GitHub and preserved with a DOI through Zenodo.
